# The fishes of Cayo Arcas (Campeche Bank, Gulf of Mexico): an updated checklist

**DOI:** 10.3897/zookeys.640.10862

**Published:** 2016-12-13

**Authors:** D. Ross Robertson, Horacio Perez-España, Enrique Nuñez Lara, Francisco Puc Itza, Nuno Simoes

**Affiliations:** 1 Smithsonian Tropical Research Institute, Balboa, Panamá; 2 Instituto de Ciencias Marinas y Pesquerías, Universidad Veracruzana, Hidalgo 617, Col. Río Jamapa, C.P. 94290, Boca del Río, Veracruz, México; 3 Facultad de Ciencias Naturales, Universidad Autónoma del Carmen, Ciudad del Carmen, Campeche, México; 4 CINVESTAV Unidad Mérida, Mérida, Yucatan, Mexico; 5 Unidad Multidisciplinaria en Docencia e Investigación de Sisal, Facultad de Ciencias, UNAM, Yucatan, México

**Keywords:** Endemic species, invasive damselfish species, reef-fishes, reef-habitat, southwest Gulf of Mexico

## Abstract

Cayo Arcas is a small, offshore reef complex on the southwest corner of Campeche Bank, Gulf of Mexico. The only published information (from 2000) on the fishes of that reef refers to 37 species. Here additional information is added, some from unpublished observations during the 1980s, as well as author observations made during 2013 and 2016. These bring the checklist of that reef’s fishes up to 162 species. The possible effects of the limited number of fish habitats available at Cayo Arcas on the composition of its fish fauna are discussed. The Indo-Pacific damselfish *Neopomacentrus cyanomos* (Bleeker, 1856) was first recorded in the Atlantic in mid-2013, on shoreline reefs in the southwest corner of the Gulf of Mexico. Recently reviewed underwater photographs show that *Neopomacentrus cyanomos* also was present at Cayo Arcas in mid-2013, 350 km from the first-record site. Hence it evidently had a substantial population in the southwest Gulf of Mexico in 2013, and must have arrived in there long before that year.

## Introduction

**Figure 1.**
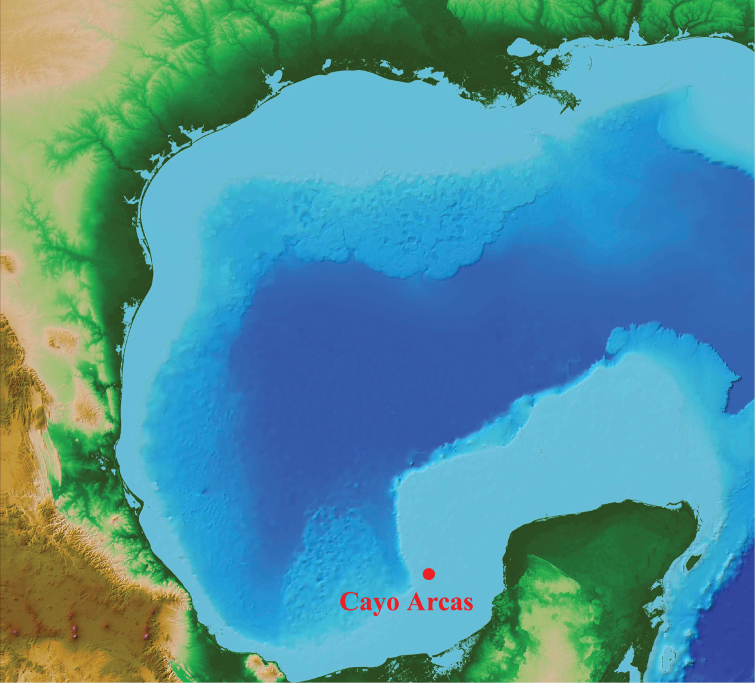
Location of Cayo Arcas in the Gulf of Mexico (Base map: NOAA).

The southwest Gulf of Mexico has relatively few coral reefs. Only a small number of these are offshore reefs on the broad, shallow Campeche Bank that extends 200+ km north from the Yucatan Peninsula. These include Alacran Reef, and a set of about 10 small submerged banks and emergent reefs scattered along the western edge of that bank (http://www.gulfbase.org/reef/). The reef-fish faunas of most of those offshore reefs are not well documented. Only two of them have substantial published checklists available: Alacran Reef, a large emergent reef in the center of the bank and the largest reef in the region (see Gonzalez-Gandara and Arias-Gonzalez 2001), and Madagascar Reef, a small, shallow, submerged rock bank 40 km offshore from Sisal (see Zarco-Perrello et al. 2014, Robertson et al. 2016). The Cayo Arcas reef complex is located near the outer edge of the southwest corner of Campeche Bank (Figure 1). Currently the only published information available on the fishes of that reef concerns 37 species that were included in an ecological study of fishes on reefs on Campeche Bank and the Mexican Caribbean by Garduño and Chavez (2000).

Here an updated checklist for the fishes of the Cayo Arcas reef complex is presented that draws on both old and new information. The object of publishing this checklist is to improve understanding of the distributions of reef fishes in the southwest Gulf of Mexico, which hosts a number of endemic reef fishes, has a different marine environment to the rest of that gulf (Belanger et al. 2012), and forms a discrete biogeographic subunit within one of three major biogeographic subdivisions of the Greater Caribbean (see Robertson and Cramer 2014).

This updated list is not complete, because the dives on which it is based did not provide comprehensive coverage of fishes across the full range of habitats available. Furthermore, because the list is based on diver observations, cryptic fishes that live within the reef matrix or within fringing soft sediments undoubtedly are under-represented. The only really effective way to comprehensively sample such fishes, which represent as much as half of any reef-associated fauna in the neotropics, is with the use of small ichthyoside stations (Robertson and Smith-Vaniz 2008). Finally, there are many fish species that include Cayo Arcas within their geographic ranges (see www.stri.org/sfgc), and that might be expected to occur there but have not yet been recorded there.

## Methods

### Study area

**Figure 2.**
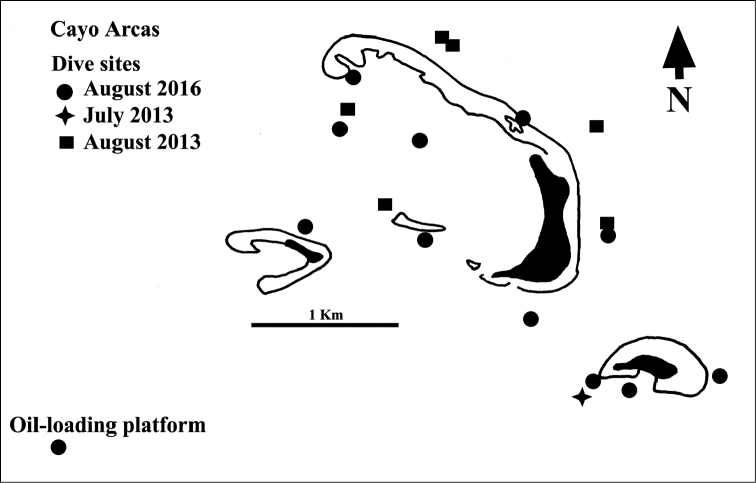
Schematic representation of Cayo Arcas reef complex. Solid lines indicate edges of emergent reef. Solid fill = sand cays. Locations of dives sites for 2013 and 2016 are indicated by symbols.

The Cayo Arcas reef complex is situated at 20.21°N, -91.98°W, 145 km from the mainland. This complex comprises reefs fringing a cluster of three sand cays that are spread over an area of ~4 km by ~2.6 km (Figure 2). The largest sand cay, which is 1 km long, has an elongate, 4 km long, crescent-shaped reef that runs along and to the northwest of its eastern side. Between the eastern beach of this cay and the exposed reef crest, there is a narrow, very shallow (~1m deep) sandy lagoon. The smaller eastern cay has a similar, much smaller lagoon (see Figure 2). On the western side of the main cay there is a large sand-floored semi-lagoon with scattered submerged patch reefs, some with abundant macroalgal growth, that gradually slopes off westwards into deeper water over a distance of ~2 km from the main cay. Reef development apparently extends down to ~25m (see http://www.gulfbase.org/reef/view.php?rid=cac1).

Mangroves and beds of seagrasses (turtlegrass, *Thalassia testudinum* Koenig 1805, and manatee-grass, *Syringodium filiforme* Kutzing in Hohenacker, 1860, represent major ancillary habitats adjacent to many reefs in the tropical northwest Atlantic. Those habitats are used by many reef fishes, often as nursery habitats. While turtlegrass often forms large beds within which dense growths of flat, strip-like blades provide ample shelter for fish, manatee-grass has thin, thread-like blades and a sparser growth pattern that provide lower quality shelter for reef fishes. There are no mangroves at Cayo Arcas. The www.gulfbase.org marine species list for reefs in the Mexican section of the Gulf of Mexico indicates that turtlegrass does occur at Cayo Arcas (see p 27 of http://www.gulfbase.org/checklist/pdfs/marine-spp-list.pdf). That gulfbase list cites Flores (1992) as the source of this information. A copy of Flores (1992) was reviewed, and it was found that, while a table that indicates what terrestrial and marine flowering plants occur at different islands shows turtlegrass as present at Cayo Arcas (and at all the other islands treated in that book), it is not mentioned in the detailed text treatment of plants at Cayo Arcas. Hence that table record may have been an error. Flores (1992) does indicate that there were no mangroves at Cayo Arcas, but does not include any mention of manatee grass at any location. Turtlegrass was not seen by us or any other divers from UNAM-Sisal studying shallow water organisms during expeditions there in April and August 2016. In contrast, manatee grass was found at Cayo Arcas during 2016, and may be restricted to the back-reef lagoon on the eastern side of the main cay, and perhaps a similar lagoon next to the eastern cay (Figure 2). In August 2016 there were windrows of dead seagrass composed exclusively of manatee-grass threads along the entire beach on the eastern side of the main cay. As both *Thalassia testudinum* and *Syringodium filiforme* are restricted to shallow water it is unlikely that turtlegrass was present but missed during the 2016 expeditions. Hence two major ancillary habitats available for reef fishes throughout much of the wider Caribbean area are entirely (mangroves) or largely (seagrass) lacking at Cayo Arcas.

### Sources of information

The earliest information available on Cayo Arcas fishes comes from an unpublished Master’s thesis by Garduño (1988). Part of this information, based on censuses of fishes made at depths between 10-42 m at Cayo Arcas during 1984-86, was published in Garduño and Chavez (2000). In 2013 HP-E censused reef fishes on twelve 25mX4m transects at one site on the Arcas reef complex on July 31, while ENL censused 24 such transects at 6-11 m depth, divided among five sites on August 6–7 (Figure 2). Most recently, during August 20-27, 2016, DRR & FPI surveyed the Cayo Arcas reefs to assess the population status and distribution of an exotic Indo-Pacific damselfish, *Neopomacentrus cyanomos* that has become established in much of the southwest Gulf of Mexico, including Cayo Arcas (see Robertson et al. 2016, Simoes and Robertson 2016). During that period 14 dives were made at 11 sites on that reef complex (Figure 2). Each dive, which ranged from 5–30 m in maximum depth, lasted 1-1.5 hrs, and covered an area of ~0.25 ha. In addition, three dives were made to depths of 40 m on a small petroleum-loading platform, located 1.5 km southwest of that reef cluster (see Figure 2, and Simoes and Robertson 2016). That platform is at the northern end of the complex of several hundred platforms that form Mexico’s largest offshore oilfield in the Gulf of Mexico. During those dives notes were made of the presence and abundance of different species of reef fishes. Due to the briefness of the 2016 visit, the primary objective of those dives was to gain a broad-brush picture of the abundance and distribution of both *Neopomacentrus cyanomos* and native fishes across a variety of habitats, rather than obtaining quantitative data on densities on small transects such as those used by HP-E and ENL.

## Results

Table 1 includes a list of 162 fish species from 41 families, mainly bony fishes, but also including three rays, together with information on their abundances, that were found at Cayo Arcas between the 1980s and 2016. During the 1980s Garduño (Garduño 1988, Garduño and Chavez 2000) recorded a total of 81 species from 28 families. In 2013 HP-E and ENL noted 78 species (including 25 additions) from 25 families. In 2016 151 species (39 families) were seen by DRR and FPI, which included 52 species not previously recorded.

**Table 1.** Preliminary checklist of fishes observed at Cayo Arcas, Campeche Bank. Abundance and distribution in 2016: Rare (1 seen during expedition); Uncommon (< 5); Occasional (~ 20, at multiple dive sites); Common, widespread (scores, at most/all dive sites); Locally common (scores to hundreds at 1–2 dive sites); Abundant, widespread (hundreds to thousands at most/all dive sites); Platform (present on oil-loading platform 1.5 km from reef complex). Abundance 2013: Mean no. fish/transect by HP-E and ENL (see methods); Abundance 1984-86: G&C 2000 = Garduño and Chavez 2000, no. individuals min^-1^ from their Table 2; G 1988 = Garduño 1988, relative abundance - mean of 10 counts).

Taxon	Abundance and distribution 2016	Abundance 2013 HP-E/ENL	Abundance 1984-86 G&C 2000/ G 1988
UROTRYGONIDAE			
*Urobatis jamaicensis*	Rare		
DASYATIDAE			
Hypanus americana*	Uncommon		0/0.1
AETOBATIDAE*			
*Aetobatus narinari*			0/0.1
MEGALOPIDAE			
*Megalops atlanticus*	Locally common (schools); platform		0/0.2
MURAENIDAE			
*Gymnothorax funebris*	Uncommon		
*Gymnothorax moringa*	Rare		0/0.1
ATHERINIDAE			
*Atherina harringtonensis* *	Locally common (large schools)		
*Atherinomorus stipes*	Locally common (large schools)		
HOLOCENTRIDAE			
*Holocentrus adscensionis*	Common, widespread	0.097/0.250	
*Holocentrus rufus*	Occasional, widespread		0/0.9
*Myripristis jacobus*	Uncommon		
*Neoniphon vexillarium*	Uncommon		
AULOSTOMIDAE			
*Aulostomus maculatus*	Uncommon	0.042/0.125	0/0.2
SCORPAENIDAE			
*Pterois volitans*	Occasional, widespread; platform (species note)		
SERRANIDAE			
*Cephalopholis cruentata*	Common, widespread; platform	0.042/0.125	0/0.2
*Cephalopholis fulva*		0.014/0.042	0/0.3
*Epinephelus adscensionis*	Common, widespread	0.055/0.083	0/1.1
*Epinephelus guttatus*	Common, widespread	0.111/ 0.333	0.05/1.0
*Hypoplectrus aberrans*	Locally common		
*Hypoplectrus ecosur*	Locally common		
*Hypoplectrus gemma*		0.014/0	
*Hypoplectrus indigo*	Uncommon		
*Hypoplectrus maculiferus*	Rare		
*Hypoplectrus nigricans*	Occasional, widespread	0.028/0.083	0/0.1
*Hypoplectrus puella*	Occasional, widespread	0.097/0.250	0/0.4
*Hypoplectrus randallorum*	Rare		
*Hypoplectrus unicolor*	Rare		
*Mycteroperca bonaci*	Common, widespread; platform		0/1.0
*Mycteroperca interstitialis*	Locally common		
*Mycteroperca microlepis*	Uncommon		
*Mycteroperca phenax*	Uncommon	0.014/0.042	
*Mycteroperca tigris*	Occasional, widespread	0.014/0.042	0/0.4
*Paranthias furcifer*	Locally common (aggregations); platform		
*Serranus baldwini*	Rare		
*Serranus tabacarius*	Locally common		
*Serranus tigrinus*	Common, widespread	0.083/0.167	0/0.7
*Serranus tortugarum*	Locally common (aggregations)		
GRAMMATIDAE			
*Gramma loreto*	Common, widespread	0.139/ 0.208	0.10/0.7
MALACANTHIDAE			
*Malacanthus plumieri*			0/0.1
OPISTOGNATHIDAE			
*Opistognathus aurifrons*	Locally common (aggregations)	0.028/0	
RACHYCENTRIDAE			
*Rachycentron canadum*	Rare; platform only		
CARANGIDAE			
*Caranx bartholomaei*	Uncommon (small schools)		
*Caranx crysos*	Common, widespread (aggregations)		0/0.3
*Caranx latus*	Common, widespread (aggregations)	0.028/0	
*Caranx lugubris*	Uncommon (aggregation); platform only		
*Caranx ruber*	Locally common (aggregations)	0.305/0.208	0.05/0.7
*Decapterus macarellus*	Locally common (school)		
*Trachinotus carolinus*	Rare		
LUTJANIDAE			
*Lutjanus analis*	Locally occasional		0/0.1
*Lutjanus apodus*	(Species note)		0.05/0.1
*Lutjanus buccanella*	Occasional (juveniles)		
*Lutjanus griseus*	Common, widespread; platform		0/1.2
*Lutjanus jocu*	Common, widespread	0.028/0.083	
*Lutjanus mahogoni*	Occasional, widespread		0/0.1
*Lutjanus synagris*	Occasional (juveniles)		
*Ocyurus chrysurus*	Abundant, widespread; platform	2.619/7.333	0.05/1.1
GERREIDAE			
*Gerres cinereus*	Uncommon	0.014/0.042	0/0.1
HAEMULIDAE			
*Anisotremus virginicus*	Locally common (school)		0.05/0
*Emmelichthyops atlanticus*	Locally common (schools)		
*Haemulon aurolineatum*	Common, widespread (aggregations)	0.014/0	0.05/0
*Haemulon carbonarium*	Uncommon		0.05/0
*Haemulon chrysargyreum*	Locally common (schools)		0.05/0.4
*Haemulon flavolineatum*	Common, widespread	1.067/1.125	0.73/2.4
*Haemulon macrostomum*	Uncommon		0/0.7
*Haemulon melanurum*	Uncommon		
*Haemulon plumierii*	Uncommon	0.083/0	0.05/0
*Haemulon sciurus*	Uncommon		0.05/0.4
*Haemulon striatum*	Locally common (large schools)		
*Haemulon vittatum* *	Locally common (large schools)	1.386/0	12.83/1.7
SPARIDAE			
*Calamus calamus*	Common, widespread	0.055/0	
*Calamus nodosus*	Common, widespread		
SCIAENIDAE			
*Equetus punctatus*	Uncommon		
*Pareques acuminatus*	Uncommon		
MULLIDAE			
*Mulloidichthys martinicus*	Common, widespread (schools)	0.014/0.083	0/2.4
*Pseudupeneus maculatus*	Locally common	0.028/0	
PEMPHERIDAE			
*Pempheris schomburgkii*	Locally common (aggregations)		
KYPHOSIDAE*		3.672/1.542	
*Kyphosus bigibbus*	Occasional		
*Kyphosus cinerascens*	Common, widespread		
*Kyphosus sectatrix*	Common, widespread		0/0.5
*Kyphosus vaigiensis*	Common, widespread		
CHAETODONTIDAE			
*Chaetodon aculeatus*			0/0.2
*Chaetodon capistratus*	Occasional		
*Chaetodon ocellatus*	Common, widespread	0.194/0.458	0/1.5
*Chaetodon sedentarius*	Common, widespread	0.152/0.250	
POMACANTHIDAE			
*Holacanthus bermudensis*	Uncommon		
*Holacanthus ciliaris*	Common, widespread; platform		0/0.4
*Holacanthus tricolor*			0.05/0
*Pomacanthus arcuatus*	Locally common	0.055/0	0/1.2
*Pomacanthus paru*	Locally common	0.014/0.042	0.05/0.5
CIRRHITIDAE			
*Amblycirrhitus pinos*	Rare; platform only		
POMACENTRIDAE			
*Abudefduf saxatilis*	Locally abundant, widespread	1.108/1.167	7.16/2.1
*Chromis cyanea*	Locally common	0.430/0	0.05/0.2
*Chromis insolata*	Locally common	0.028/0.083	0/0.2
*Chromis multilineata*	Abundant, widespread	47.056/ 90.750	28.93/3.2
*Chromis scotti*	Common, widespread; platform	0.194/0	
*Microspathodon chrysurus*	Common, widespread	0.152/0.375	1.14/2.3
*Neopomacentrus cyanomos*	Abundant, widespread (aggregations); platform. (species note)	Present/0	
*Stegastes adustus*	Abundant, widespread (species note)	0.443/0.167	
*Stegastes diencaeus*		0.055/0.167	1.14/1.4
*Stegastes leucostictus*	Locally common	0.222/0.417	0/0.3
*Stegastes partitus*	Abundant, widespread; platform	3.603/4.667	0.05/0.9
*Stegastes planifrons*	Abundant, widespread	0.679/1.500	12.93/3.7
Stegastes xanthurus*	Abundant, widespread (species note)	0.679/0.542	0.05/0
LABRIDAE			
*Bodianus pulchellus*	Locally common; platform only		
*Bodianus rufus*	Common, widespread	0.651/1.208	0/1.1
*Clepticus parrae*	Locally common, schools	1.261/3.792	9.73/0.4
*Halichoeres bivittatus*	Very common, widespread	0.443/0.083	0/0.9
*Halichoeres burekae*	Abundant, widespread (species note)	5.085/0.083	
*Halichoeres garnoti*	Common, widespread	2.245/4.452	0.05/0.2
*Halichoeres maculipinna*	Common, widespread	0.402/1.208	1.36/1.3
*Halichoeres poeyi*		0.319/0	
*Halichoeres radiatus*	Common, widespread	0.222/0.083	0/1.0
*Lachnolaimus maximus*	Uncommon		
*Thalassoma bifasciatum*	Abundant, widespread; platform	10.752/11.833	1.22/2.1
*Xyrichtys splendens*	Uncommon; local		
SCARIDAE			
*Cryptotomus roseus*	Occasional		
*Scarus coelestinus*	Occasional		0/0.6
*Scarus coeruleus*	Common, widespread	0.152/0	0.05/1.6
*Scarus guacamaia*	Occasional, widespread	0.042/0	0/0.3
*Scarus iseri*	Common, widespread	2.480/3.042	
*Scarus taeniopterus*	Common, widespread	0.111/0.333	5.40/1.3
*Scarus vetula*	Common, widespread	1.178/2.875	5.23/3.4
*Sparisoma atomarium*	Locally common	0.055/0.167	
*Sparisoma aurofrenatum*	Common, widespread	1.857/2.833	0/0.4
*Sparisoma chrysopterum*	Occasional, widespread	0.014/0.042	0.05/1.7
*Sparisoma radians*	Common, widespread		
*Sparisoma rubripinne*	Locally common	0.291/0.500	0/1.5
*Sparisoma viride*	Very common, widespread	1.136/1.833	3.63/3.2
TRIPTERYGIIDAE			
*Enneanectes boehlkei*	Present (species note)		
BLENNIIDAE			
*Entomacrodus nigricans*	Uncommon; platform only		
*Hypsoblennius invemar*	Locally common; platform only		
*Ophioblennius macclurei* *	Common, widespread	0.028/0	0/01.2
*Parablennius marmoreus*	Locally common; platform		
*Scartella cristata*	Locally common; platform only		
LABRISOMIDAE			
*Malacoctenus aurolineatus*	Locally common		
*Malacoctenus macropus*	Common, widespread		
*Malacoctenus triangulatus*	Common, widespread	0.097/0.083	
*Starksia ocellata*	Present (species note)		
GOBIIDAE			
*Coryphopterus dicrus*	Abundant, widespread		
*Coryphopterus glaucofraenum*	Abundant, widespread	0.319/0	
*Coryphopterus hyalinus*/*personatus*	Abundant, widespread (species note)	4.432/5.625	
*Elacatinus oceanops*	Common, widespread	0.222/0.292	
*Elacatinus xanthiprora*	Uncommon		
*Gnatholepis thompsoni*	Abundant, widespread	0.249/0.042	
PTERELEOTRIDAE			
*Ptereleotris calliura*	Locally common (aggregations)		
ACANTHURIDAE			
*Acanthurus chirurgus*	Common, widespread	0.097/0.167	0.05/0.8
*Acanthurus coeruleus*	Common, widespread	0.139/0.250	0.05/1.6
*Acanthurus tractus* *	Common, widespread (species note)	0.291/0.875	1.09/1.2
SPHYRAENIDAE			
*Sphyraena barracuda*	Occasional, widespread	0.014/0.042	0/1.1
BALISTIDAE			
*Balistes capriscus*	Rare		
*Balistes vetula*		0.042/0.125	
*Canthidermis sufflamen*	Occasional, widespread		0/0.1
*Melichthys niger*	Locally common		0/0.2
*Xanthichthys ringens*			0/0.2
MONACANTHIDAE			
*Aluterus scriptus*	Occasional, widespread		0/0.3
*Cantherhines pullus*	Rare	0.028/0	
*Monacanthus tuckeri*	Locally common	0.028/0	
OSTRACIIDAE			
*Lactophrys bicaudalis*	Rare		0/0.1
*Lactophrys triqueter*	Occasional, widespread	0.014/0.042	0/0.8
TETRAODONTIDAE			
*Canthigaster rostrata*	Common, widespread	0.152/0.250	0.05/0.8
*Sphoeroides testudineus*	Rare		
DIODONTIDAE			
*Diodon hystrix*	Uncommon		0/0.4

* Taxonomic notes: *Hypanus americana*: this species has been moved from the genus *Dasyatis* to *Hypanus* (see Last et al. 2016). AETOBATIDAE: This family was recently resurrected by White and Naylor (2016). *Atherina harringtonensis*: Previously known as *Hypoatherina harringtonensis*, this was reclassified as a species of *Atherina* by Sasaki and Kimura (2014). *Haemulon vittatum*: Once known as *Inermia vittata*, this has been shown to be a *Haemulon* species (see Rocha et al. 2008). *Kyphosus* species: Due to morphological similarities and inadequate knowledge of the number and identity of species potentially present in the Greater Caribbean, members of this genus there often have not been accurately identified to species; furthermore, the older literature on Greater Caribbean kyphosids only mentions *Kyphosus sectatrix* and *Kyphosus incisor*. However, the genus recently was comprehensively revised by Knudsen and Clements (2013, 2016), who identified four species in that area: *Kyphosus bigibbus*, *Kyphosus cinerascens*, *Kyphosus sectatrix* and *Kyphosus vaigiensis* (see http://biogeodb.stri.si.edu/caribbean/en/gallery/genus/1571). Accurate descriptions of field characteristics of those four, an extensive database of photographs of them that were identified by those authors, and careful, close-range inspection of fish in the field now enable identification of members of this genus at different locations in that region. All four species were present at Cayo Arcas in 2016. *Stegastes xanthurus*: Garduño and Chavez (2000), HP-E and ENL all recorded *Stegastes variabilis* as present at Cayo Arcas. However, *Stegastes variabilis* is restricted to Brazil, and genetically distinct from the Caribbean form, *Stegastes xanthurus. Acanthurus tractus*: This was previously recorded as *Acanthurus bahianus*, which is restricted to Brazil. *Acanthurus tractus* is its sister species in the Greater Caribbean. *Ophioblennius macclurei*: Often recorded in the older literature as *Ophioblennius atlanticus*, which is now known to be restricted to the eastern Atlantic.

### Species Notes

***Pterois volitans*** (Linnaeus, 1758). This invasive Indo-Pacific lionfish was first reported on the USGS invasive species website at an oil platform near Cayo Arcas in late 2012 (http://nas.er.usgs.gov/queries/SpecimenViewer.aspx?SpecimenID=292473. Fewer than a dozen individuals of this species were seen by the group of eight divers during the August 2016 expedition. Those occurred at depths of between 8-20 m on the reef, and at 30 m on the oil platform. Lionfish were first reported at Alacran reef, 350 km northeast of Cayo Arcas, in 2010, and on coastal reefs ~450 km west of Cayo Arcas in 2012 (http://nas.er.usgs.gov/queries/collectioninfo.aspx?SpeciesID=963). At Alacran Reef lionfish seem to be concentrated at mesophotic depths (Aguilar-Perera et al. 2016). It is not known how abundant lionfish is in the Cayo Arcas area below 25 m depth.

***Hypoplectrus nigricans*** (Poey, 1852). This species is known to vary geographically in color and shape (Aguilar-Perera 2004). At Cayo Arcas in 2016 all *Hypoplectrus nigricans* were similarly colored, with a translucent, dark blue-black head, body and fins, except the tail fin, which was translucent grey, with darker upper and lower borders.

***Lutjanus apodus*** (Walbaum, 1792). Garduño (1988) Garduño and Chavez (2000) listed only two snappers, *Lutjanus apodus* and *Ocyurus chrysurus* (Bloch, 1791) as present at Cayo Arcas. The seven members of this family that were observed during 2016 included six species of *Lutjanus*, but not *Lutjanus apodus*. *Lutjanus apodus* also was not recorded in 2013. Two of those *Lutjanus* species, *Lutjanus griseus* (Linnaeus, 1758) and *Lutjanus jocu* (Bloch & Schneider, 1801) were common across on the Arcas reefs in 2016. *Lutjanus apodus*, which is easily recognizable and probably the commonest and most widely distributed shallow-living member of its genus on Caribbean reefs, occurs through much of the Gulf of Mexico (see http://www.iucnredlist.org/details/155152/0, and http://biogeodb.stri.si.edu/caribbean/en/thefishes/species/3684. If currently present in the Arcas reef complex *Lutjanus apodus* must be rare.

**Figure 3.**
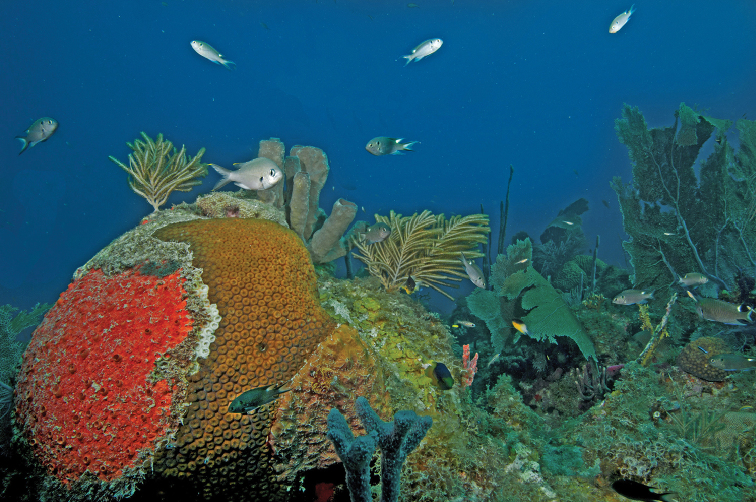
*Neopomacentrus cyanomos* at Cayo Arcas, July 2013 (Photo HP-E); 11 *Neopomacentrus cyanomos* are visible, and can be identified by the presence of a black blotch on the shoulder, and a large white blotch on the rear edge of the dorsal fin; a single *Chromis multilineata* (without the shoulder- and dorsal-fin blotches) is present in the foreground immediately above the brain coral.

***Neopomacentrus cyanomos***. During a visit to Cayo Arcas in April 2016 NS found it to occur both on the reefs and the adjacent oil-loading platform, where it was superabundant (Simoes and Robertson 2016). During the August 2016 expedition we found this species in small aggregations (up to ~150 fish, but usually about several dozen, including many juveniles) at all dive sites on the reef complex. It also formed a large, dense aggregation of many tens of thousands of individuals on the oil-loading platform (see Simoes and Robertson 2016). Neither HP-E nor ENL recorded this species in 2013. However, a 2016 review by HP-E of photographs he took during his July 2013 expedition show that this species was present on the Cayo Arcas reef then (Figure 3).

***Stegastes adustus*** (Troschel in Muller, 1865) and ***Stegastes diencaeus*** (Jordan & Rutter, 1897). In 2016 *Stegastes adustus*, a benthic-feeding, omnivorous damselfish, was common on all hard reef substrata not covered by live corals, at depths of 0.5-7 m. HP-E recorded *Stegastes adustus* but not *Stegastes diencaeus*, while ENL recorded both species. In their list Garduño and Chavez 2000 did not include either species by name, but did record *Eupomacentrus mellis* Emery & Burgess, 1974 which is the juvenile of *Stegastes diencaeus* (see Robertson and Allen 1981). Adults of both these *Stegastes* species are dull brown to blackish fishes with similar shapes and body sizes that are often confused, even in guide books by experts: e.g. figure 287 of Randall (1996) is *Stegastes diencaeus*, not *Stegastes adustus*; the lower photo on p. 171 of Allen (1991) is *Stegastes diencaeus*, not *Stegastes adustus*; and the image on the bottom of p. 113 of Humann and DeLoach (1994) is *Stegastes diencaeus*, not *Stegastes adustus* (an error that was corrected in later editions of that book). In addition, juvenile *Stegastes diencaeus* displaying the “mellis” coloration look quite similar to juveniles of two other *Stegastes* species commonly found at Cayo Arcas: *Stegastes leucostictus* (Muller and Troschel in Schomburgk 1848) and *Stegastes xanthurus* (Poey, 1860; recorded as *Stegastes variabilis* (Castelnau, 1855) by Garduño and Chavez (2000); see http://biogeodb.stri.si.edu/caribbean/en/gallery/specie/3879, and http://biogeodb.stri.si.edu/caribbean/en/gallery/specie/3884). Given the abundance of *Stegastes adustus* at Cayo Arcas in August 2016 and the apparent absence of *Stegastes diencaeus* then, and the similarities between adults and juveniles of *Stegastes diencaeus* and its congeners, differences in those *Stegastes* spp recorded during and prior to 2016 might have been due to misidentifications. In contrast, both *Stegastes adustus* and *Stegastes diencaeus* were recorded at Alacran Reef by González-Gándara and Arias González (2001, 2004) and both were commonly observed by DRR during two weeks of diving there in May 2016.

***Halichoeres burekae*** Weaver & Rocha, 2007. This recently described species is endemic to the southwest and northwest Gulf of Mexico. Its known range extends from Alacran Reef on Campeche Bank to the Flower Garden Banks at the edge of the continental shelf off Texas (see http://biogeodb.stri.si.edu/caribbean/en/thefishes/species/4707; and http://maps.iucnredlist.org/map.html?id=187608). At Alacran Reef, 350 km northeast of Cayo Arcas, Aguilar-Perera and Tuz-Sulub (2009) described this species as occurring in small aggregations of a few dozen fish to as many as 200 fish, and that it is widely distributed there. Observations of the abundance and distribution of *Halichoeres burekae* at Alacran Reef made by DRR during May 2016 are consistent with those of Aguilar-Perera and Tuz-Sulub (2009). This species is relatively uncommon at the Flower Gardens area (http://www.iucnredlist.org/details/187608/0). During August 2016 *Halichoeres burekae* was recorded at all dive sites on the Cayo Arcas reefs (but not the oil-loading platform) and was one of the most abundant labrids on that reef complex. It was at least an order of magnitude more abundant than observed by DRR at Alacran Reef during June 2016. Multiple aggregations of scores to hundreds of individuals moving about in the water column 1-3m above the bottom were seen at each dive site. Those aggregations were mainly seen in areas of low relief, over both sandy and coralline-rock bottoms. A large aggregation containing thousands of individuals was seen mass spawning in midwater over a substratum of dense, live gorgonian trees on the fore-reef off the center of the main-cay reef at ~10m depth during one afternoon dive.

***Scarus guacamaia*** Cuvier, 1829. At Cayo Arcas one or two adults of this species were seen on each of eight of the 14 dives on the reef complex in 2016. *Scarus guacamaia* also was recorded by Garduño 1988, and by HP-E in 2013. This species has been thought to be dependent on mangroves as habitat for juveniles (http://www.iucnredlist.org/details/19950/0). However Cayo Arcas lacks mangroves, and the nearest mangroves are located on the coast, 145 km away. Although <20 *Scarus guacamaia* were seen at Cayo Arcas in 2016, a more comprehensive survey across a broad range of habitats would be needed to provide a reliable estimate its total population size there.

***Enneanects boehlkei*** Rosenblatt, 1960 and ***Starksia ocellata*** (Steindachner, 1876). One individual of each of these two cryptic species was collected as bycatch while using clove oil to anaesthetize *Neopomacentrus cyanomos* for collection.

***Coryphopterus hyalinus*** Bohlke & Robins, 1962, and ***Coryphopterus personatus*** (Jordan & Thompson, 1905). This pair of sister species (see Baldwinet al. 2009) apparently have essentially the same external appearance, and are differentiated by the patterns of pores on the top of the head. They also have very similar geographic, habitat and depth ranges (see http://biogeodb.stri.si.edu/caribbean/en/thefishes/species/4119 and http://biogeodb.stri.si.edu/caribbean/en/thefishes/species/4121). We include them as a single unit as, while diving, we were unable to determine whether one or both occurs at Cayo Arcas.

## Discussion

The present checklist includes 162 species from 41 families. The great majority of those species are widespread in the Greater Caribbean, with only *Halichoeres burekae* and *Hypoplectrus gemma* Goode and Bean, 1882 representing species that are entirely (or almost so) restricted to the Gulf of Mexico. Most of the geographically widely distributed species that were common in the 1980s and 2013 surveys at Cayo Arcas also were common there in 2016. There are several noteworthy features of the suite of species found at Cayo Arcas: *Chromis multilineata* (Guichenot, 1853), which is a common species widely distributed on reefs throughout the Greater Caribbean, was notably abundant in all surveys made between the 1980s and 2016. *Halichoeres burekae*, a western Gulf of Mexico endemic, was (perhaps) the most common labrid at Cayo Arcas, and was more abundant there than has been recorded anywhere else previously. This species is listed as Endangered (i.e. at a high risk of extinction) by the IUCN Red List (http://www.iucnredlist.org/details/187608/0), due to the small size of its geographic range and the paucity of reef habitat in that range. The abundance of this species at Cayo Arcas has substantial conservation significance for this species, as it indicates that the set of small offshore reefs scattered along the western side of Campeche Bank may be essential for its continued existence. None of those reefs is a yet designated a marine protected area.

*Neopomacentrus cyanomos* was first recorded in the Atlantic by González-Gándara and Cruz-Francisco (2014). In mid-2013 those authors found this species to be common on shoreline reefs near Coatzacoalcos, in the extreme southwest corner of the Gulf of Mexico. In 2014-15 it was found to occur more widely, on center-shelf and shoreline reefs between Madagascar Reef (near Sisal, Yucatan) and Tuxpan, Veracruz state (Cruz-Francisco et al. 2016, Robertson et al. 2016). Figure 3 here demonstrates that its range already spanned at least 350 km when it was first discovered in the Gulf of Mexico. Hence it likely arrived in the Gulf of Mexico long before 2013.

Two species that are commonly found on reefs nearby and more widely within the greater Caribbean were conspicuously absent/rare at Cayo Arcas: *Lutjanus apodus* and *Stegastes diencaeus*. *Lutjanus apodus* is one of the species thought to be strongly dependent on mangroves as nursery habitat in the Caribbean area (Nagelkerken et al. 2001, Halpern 2004, Naglekerken 2009). Although there are no mangroves at Cayo Arcas, various other species that make strong usage of mangroves as nursery habitat (Nagelkerken et al. 2001, Naglekerken 2009) are present and not rare at Cayo Arcas (*Lutjanus griseus*, *Lutjanus mahogoni* (Cuvier, 1828), *Ocyurus chrysurus*, *Haemulon flavolineatum* (Desmaret, 1823), *Sparisoma chrysopterum* (Bloch and Schneider, 1801), and *Sphyraena barracuda* (Edwards, 1771)), while others are absent to uncommon (*Haemulon sciurus* (Shaw, 1803), *Chaetodon capistratus* Linnaeus, 1758, *Gerres cinereus* (Walbaum, 1792)). Among the species that Nagelkerken (2009) indicates make major use of seagrass as nursery habitat some were common (*Acanthurus chirurgus* (Bloch, 1787), *Scarus coeruleus* (Edwards, 1771), *Scarus iseri* (Bloch, 1798), and *Ocyurus chrysurus*), but not all (*Haemulon plumieri* (Lacepede, 1801)). The extent to which manatee-grass and macroalgae can fill the nursery role of turtlegrass beds for some reef fishes clearly needs assessment. While lack of such habitats at Cayo Arcas may account for the absence of *Lutjanus apodus*, the paucity of *Stegastes diencaeus* cannot so readily be explained, as this species is common at Alacran Reef, 350 km northeast of Cayo Arcas, and it occurs on coastal reefs to the west of Cayo Arcas (see Del Morales-Floreset al. 2013). Variation in species occurrences such as these show that small, isolated offshore reefs such as Cayo Arcas that have a limited range of habitats offer considerable potential for testing ideas about ecological determinants of the abundance and geographic distributions of Greater Caribbean reef fishes.

Future, comprehensive faunal surveys should be made of the reef fish faunas not only of Cayo Arcas but also other, more poorly known emergent reefs and submerged banks near the outer edges of Campeche Bank to assess their reef fish faunas, to gain a better understanding of the biogeography of their fishes, and their importance for conservation, as well as assessing them as natural biogeographical experiments. Those surveys should also involve genetic analyses to determine the extent to which the regional fauna and faunas of individual reefs include cryptic endemic species.
